# Determining the Educational Value of an Emergency Medicine Rotation for Non-Emergency Medicine Residents

**DOI:** 10.7759/cureus.47284

**Published:** 2023-10-18

**Authors:** Carolina Veronese, Matthew Williams, Jacob Dickson, Montika Bush, Christina Shenvi

**Affiliations:** 1 Emergency Medicine, Alamance Regional Medical Center, Burlington, USA; 2 Emergency Medicine, Twin Cities Hospital, Paso Robles, USA; 3 Emergency Medicine, Texas Health Resources, Plano, USA; 4 Emergency Medicine, University of North Carolina at Chapel Hill School of Medicine, Chapel Hill, USA; 5 Emergency Medicine, University of North Carolina (UNC) at Chapel Hill, Chapel Hill, USA

**Keywords:** medical resident education, bedside learning, emergency medicine resident, teaching in emergency medicine, emergency medicine training

## Abstract

Background

Residents from diverse specialties perform clinical rotations in the emergency department (ED). There is little research about the value of the ED rotation for them.

Objectives

We sought to determine the learning objectives of non-EM residents (NEMRs) in the ED, the effectiveness of the rotation, and the highest-yield components of their experience.

Methods

From 2017-2019, we surveyed NEMR on their pre-rotation learning objectives and their comfort level with 15 common ED presentations/procedures before and after the rotation. We assessed how well their objectives were met, the highest-yield components of their rotation, and opportunities for improvement.

Results

We collected responses from 56 (47%) pre-rotation and 61 (51%) post-rotation residents over a two-year period. The five most commonly cited learning goals were: management of acutely ill patients, triage skills, procedural competence, and ultrasound. Seventy-eight percent (78%) of residents reported their learning goals were moderately to very well met during their rotation. NEMRs’ level of comfort improved in all the commonly encountered clinical experiences in the ED in a statistically significant manner. They cited on-shift teaching by attending physicians and senior EM residents as the most valuable learning resource.

Conclusion

NEMR from diverse medical and surgical specialties could identify specific learning objectives for their EM rotation with common themes, and the majority felt their educational goals were met. They gained comfort with the management and triage of all the assessed common ED conditions. By collecting and defining their specific needs and goals, we are better equipped to improve the quality and value of the rotation.

## Introduction

The emergency department (ED) affords a unique learning environment encompassing acute, critical, and primary care of the undifferentiated patient. Many medical, surgical, and psychiatry residency programs require clinical rotations in the ED, some of which are mandated by the Residency Review Committee (RRC) [1]. The Accreditation Council for Graduate Medical Education (ACGME) outlines some very general objectives for NEMRs. For example, objectives related to emergency care from the internal medicine guidelines include: “recognize and provide initial management of emergency medical problems,” and that residents must demonstrate that they can manage patients in “the inpatient ward, the critical care units, the emergency setting, and the ambulatory setting” [2]. In 2007, the American College of Emergency Physicians suggested a curriculum for non-emergency medicine (EM) residents (NEMRs) rotating in the ED [3]. More recently, a few surveys have been published that further define current educational gaps for NEMRs. In two small, prior studies, NEMRs reported that they felt ill-prepared to manage a number of emergency conditions prior to the EM rotation [4] but also did not always meet their own identified learning goals during the rotation [5].

Despite the widespread use of the ED as a learning environment for non-EM residents (NEMR), very little work has been done to determine the specific educational needs and objectives of residents and the effectiveness of the rotation [6].

We sought to improve the experience and value of the ED rotation for NEMRs by first understanding their learning objectives and how well they are being met at our institution. Rather than assess the residents based on the ACGME-delineated objectives, we framed our pilot needs assessment study in the context of the residents as master adaptive learners [7-9]. In this model, adult learners are able to assimilate and integrate knowledge and develop adaptive expertise when they go through a four-stage process of (1) identifying gaps in knowledge, skills, or attitudes, (2) developing their own learning objectives and seeking experiences or resources to fill those gaps, (3) assessing their new skills or knowledge by trying it out or discussing it with others, and finally, (4) adjusting their practice based on what they have learned [7,10]. By querying them to identify their learning objectives for the rotation, they are performing the first two steps in this adaptive process.

## Materials and methods

This study was performed in a single, academic tertiary care center in the Southeast and was granted IRB exemption. Approximately 55-60 NEMRs rotate in the ED for four-week periods annually. The NEMRs include a mix of post-graduate year (PGY)-1 and PGY-2 residents from six different specialties.

Over the course of a 24-month period, NEMRs were sent pre- and post-rotation surveys. The pre-rotation survey assessed the residents’ current level of medical training, prior EM experience, and level of comfort with a set of previously identified common clinical conditions and procedures seen in the ED [11] based on a five-point Likert scale. We also used open-ended questioning to allow each respondent to identify their individual top three learning objectives for their rotation, which were then grouped into similar categories and analyzed qualitatively. Inclusion criteria included all non-EM residents performing their required EM rotation in the ED during the 24-month study period. No residents were excluded.

During the rotation, the residents completed 17-19 clinical shifts in the ED. They attended at least one five-hour session of resident conference didactics and were provided with an online curriculum of learning modules and a podcast series. They received ultrasound teaching on shift by the attending and senior EM residents in a non-structured way based on individual patient care. The post-rotation survey assessed their level of comfort with the same clinical conditions.

For quantitative responses, descriptive statistics, including means and error ranges, were calculated. The weighted average of the five-point Likert scale for residents’ comfort level with 15 core EM presentations was calculated from pre- and post-rotation responses and compared using T-tests to determine the statistical significance of mean differences using SAS software (Cary, NC) version 9.4 or greater. Means and 95% confidence limits for each weighted average were summarized by clinical condition. Two-sample T-tests assumed that respondents were from independent samples with unequal group variances since we did not collect a unique identifier to match pre-/post- survey responses. All authors were employed by the study institution during the project timeline.

## Results

Pre- and post-rotation surveys were sent to 119 NEMRs. The total number of participants who completed surveys included 56 pre-rotation (47%) and 61 post-rotation surveys (51%). A summary of the survey participants’ level of training, specialty, and prior EM experience is presented in Table 1. The most cited educational goals included a desire to learn about the management of acutely or critically ill patients, triage skills, procedural competence, and ultrasound training (Table 2).

**Table 1:** Summary of non-emergency medicine residents’ level of training, specialty, and prior emergency medicine experience

Specialty		Prior EM Experience (all applicable)		Post Graduate Year	
Internal Medicine	18 (29%)	Clerkship (3^rd^ yr)	20 (32%)	PGY-1	46 (74%)
Family Medicine	18 (29%)	Sub-internship (4^th^ yr)	27 (43%)	PGY-2	16 (26%)
Medicine/Pediatrics	8 (13%)	No prior EM rotations	22 (35%)		
PMR	7 (11.3%)				
Orthopedics	4 (6.5%)				
Neurology	4 (6.5%)				
ENT	3(4.8%)				

**Table 2:** Most frequently reported educational goals and learning objectives identified by non-emergency medicine residents prior to their rotation in the emergency department

Learning Goal	% of NEMR
Triage skills	28%
Management of acute or critically ill patients	23%
Procedural skills	16%
Ultrasound	15%
Presentation skills	5%
Trauma	5%
Other	8%

**Figure 1:**
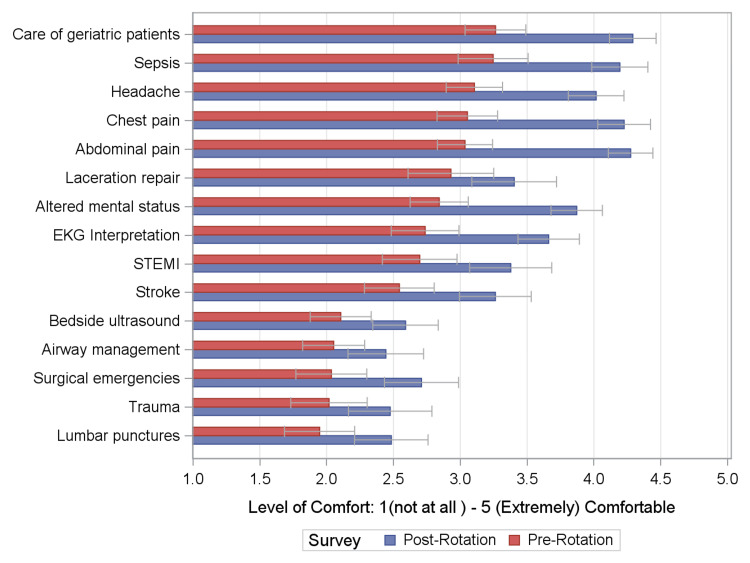
Comparison of non-emergency medicine residents’ level of comfort with 15 core emergency medicine presentations, populations, or procedures before and after the rotation The values shown are the mean level of comfort noted among all participants before (gray) and after (black) the rotation. For all changes, p<0.04. Error bars are indicated.

After the rotation, the residents reported statistically significantly (p<0.04 for all) higher levels of comfort with all the identified core ED clinical conditions and skills (Figure 1). Comfort levels increased most in the management of chest pain and abdominal pain, and least in the management of trauma and airway. These latter two are expected, as the NEMRs do not primarily manage trauma activations or perform intubations. The majority of NEMRs surveyed (78%) reported that their educational goals were moderately or very well achieved by the end of their rotation.

**Figure 2:**
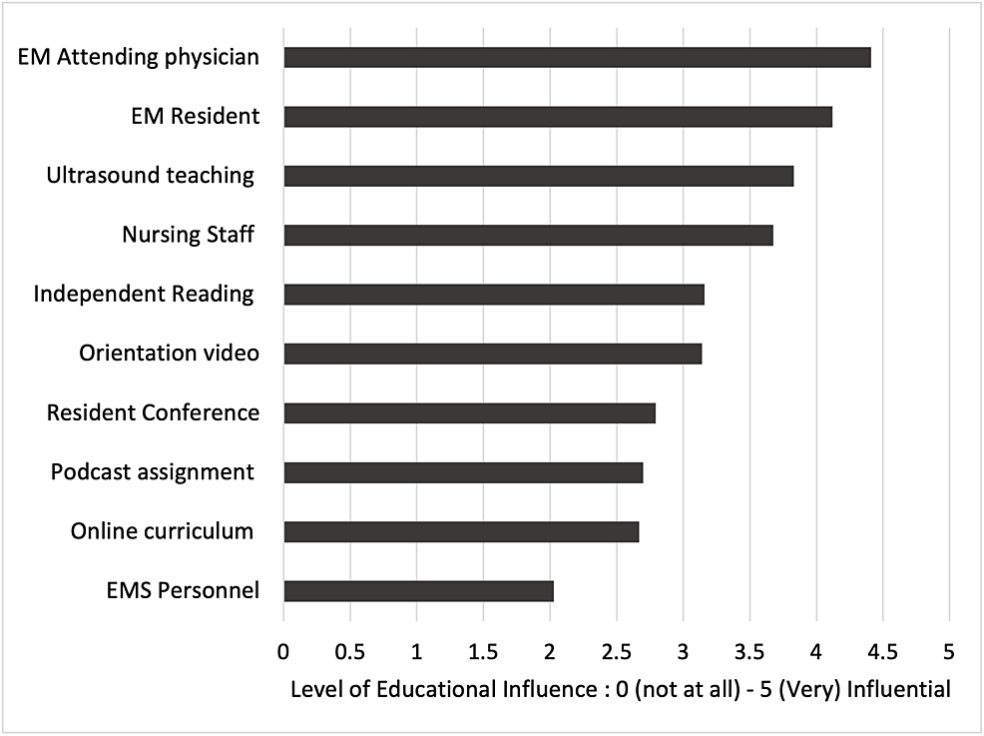
Perceived educational value of learning resources and modalities available to non-EM residents during their rotation

On-shift teaching by EM attending physicians and senior residents was the top two most influential learning resources for NEMRs (Figure 2). The two most commonly cited areas for improvement of the rotation included: a more robust orientation and improved ultrasound teaching (Table 3).

**Table 3:** Most commonly reported areas for improvement of our non-emergency medicine resident rotation in the emergency department Others* noted: expanded learning resources, documentation instruction, feedback/evaluation

Area of Improvement	Responses
Pre-rotation orientation	17 (27%)
Ultrasound teaching	13 (21%)
Trauma experience	10 (16%)
Procedural experience	9 (15%)
Scheduling	7 (11%)
Other*	6 (10%)

## Discussion

This study was an assessment at a single institution to explore the learning objectives of NEMRs in the ED and whether they are being met. The study enrolled NEMRs from diverse fields and found that the majority (78%) felt their educational goals were being met during their rotation in the ED. After the ED rotation, the NEMR’s comfort level with all the identified ED clinical conditions and procedures improved (p-value <0.04 for all conditions). Comfort level improved most in areas of commonly encountered ED conditions or populations, such as abdominal pain, chest pain, or the care of geriatric patients, and procedures such as EKG interpretation.

The NEMRs identified EM-attending physicians and senior residents as the most valuable educational resources while online training modules were the least useful and were generally not utilized. The value of on-shift teaching provides the impetus to perform further faculty and resident development to improve their teaching skills.

This study differs from and expands on work from prior studies in that it assessed residents from all specialties, not just one discipline [5], and had a larger sample size [6]. Other studies have helped define the learning objectives for NEMRs in the ED [3]. However, since learning objectives may vary significantly based on the resident's discipline, this study takes a learner-centered approach with a combination of open-ended and quantitative questions to allow more learner-driven opportunities to develop learning goals. This study also assessed the change in the comfort level of residents with different patient chief concerns as well as procedural skills.

There are several limitations to this study. It is a single-center, exploratory needs assessment, and response rates were low. Whether learning objectives were met was self-assessed by the learner. The lack of a means to link residents’ pre- and post-rotation responses limits the appropriateness of the statistical test performed. Analysis incorporating the correlation of respondent responses would be a more appropriate analysis method for drawing conclusions about changes in comfort level.

A larger study that incorporates learning objectives defined by the learner, as well as objectives identified by program directors and the ACGME, would provide a more holistic view of the value of the EM rotation. Finally, developing a more robust means of assessing whether the objectives have been met would provide stronger evidence, rather than using resident comfort level.

## Conclusions

This study demonstrated that resident learners across diverse fields have specific learning objectives for their EM rotation, and several common themes and objectives emerged. In contrast to some prior studies, in our context, those learning objectives were largely being met. In general, there is a paucity of data on this topic. This study adds a single institution’s perspective on the experience of NEMRs and the role of the EM rotation.

Future work could use learning objectives from more sources and more objective measures of residents’ competence over the course of the rotation. In addition, with a larger sample size, it may be possible to determine which aspects of the rotation were of greatest value for residents from different specialties. Furthermore, it would be interesting to determine whether the ED rotation provided a better learning opportunity during the PGY-1 vs PGY-2 year, to further optimize the residents' learning experience.

## Appendices

Off-service resident pre-rotation survey

Dear Off-Service Resident,

The purpose of this survey is to help identify your goals for the rotation in the Emergency Department. This will help us improve the rotation experience. Your responses will be kept confidential.1. What year are you?

PGY1 (intern)

PGY2 (second year)

2. In what specialty are you?

Internal Medicine

Family Medicine

Medicine/Pediatrics combined program

Orthopedics

ENT

Neurology

Physical Medicine and Rehab

Preliminary or Transitional

If you are in a preliminary or transitional year, please specify intended final specialty.

3. During medical school, did you do any of the following (check all that apply)

Emergency Medicine clerkship (during 3yd year of medical school)

Emergency Medicine sub-internship or acting internship (during 4th year of medical school)

No Emergency Medicine rotations prior to internship

4. List the top three things you want to learn during your month-long rotation in the Emergency Department

5. How comfortable do you feel with the following patients, presentations, or skills: (1 - Not at all comfortable, 2 - Slightly comfortable, 3 - Moderately comfortable, 4 - Very comfortable, 5 - Extremely comfortable)

Geriatric patients (age 65 and older)

Trauma patients

Patients with undifferentiated chest pain

Patients with undifferentiated abdominal pain

Patients with altered mental status

Patients with a stroke

Patients with a STEMI

Patients with headache

Airway management

Surgical emergencies

Sepsis

Bedside ultrasound

Performing laceration repair

Performing lumbar punctures

Interpreting EKGs

6. What types of resources do you find yourself using the most to supplement your learning now that you are a resident?

Books

Online (websites, blogs, etc...)

Articles

Podcasts

Lectures

Bottom of Form

Off-service resident post-rotation survey

Dear Off-Service Resident,

The purpose of this survey is to help identify what you learned during your rotation in the Emergency Department. This will help us improve the rotation experience. Your responses will be kept confidential.

1. What year are you?

PGY1 (intern)

PGY2 (second year)

2. In what specialty are you?

Internal Medicine

Family Medicine

Medicine/Pediatrics combined program

Orthopedics

ENT

Neurology

Physical Medicine and Rehab

Preliminary or Transitional

If you are in a preliminary or transitional year, please specify intended final specialty.

3. During medical school, did you do any of the following (check all that apply)

Emergency medicine clerkship (during 3rd year of medical school)

Emergency Medicine sub-internship or acting internship (during 4th year of medical school)

No Emergency Medicine rotations prior to internship

4. List the top three most important things you learned during your month-long rotation in the Emergency Department

5. How comfortable do you feel with the following patients, presentations, or skills: (1 - Not at all comfortable, 2 - Slightly comfortable, 3 - Moderately comfortable, 4 - Very comfortable, 5 - Extremely comfortable)

Off-service Resident Pre-Rotation Survey

Dear Off Service Resident,

The purpose of this survey is to help identify your goals for the rotation in the Emergency Department. This will help us improve the rotation experience. Your responses will be kept confidential.1. What year are you?

PGY1 (intern)

PGY2 (second year)

2. In what specialty are you?

Internal Medicine

Family Medicine

Medicine/Pediatrics combined program

Orthopedics

ENT

Neurology

Physical Medicine and Rehab

Preliminary or Transitional

If you are in a preliminary or transitional year, please specify intended final specialty.

3. During medical school, did you do any of the following (check all that apply)

Emergency Medicine clerkship (during 3yd year of medical school)

Emergency Medicine sub-internship or acting internship (during 4th year of medical school)

No Emergency Medicine rotations prior to internship

4. List the top three things you want to learn during your month-long rotation in the Emergency Department

5. How comfortable do you feel with the following patients, presentations, or skills: (1 - Not at all comfortable, 2 - Slightly comfortable, 3 - Moderately comfortable, 4 - Very comfortable, 5 - Extremely comfortable)

Geriatric patients (age 65 and older)

Trauma patients

Patients with undifferentiated chest pain

Patients with undifferentiated abdominal pain

Patients with altered mental status

Patients with a stroke

Patients with a STEMI

Patients with headache

Airway management

Surgical emergencies

Sepsis

Bedside ultrasound

Performing laceration repair

Performing lumbar punctures

Interpreting EKGs

6. Which of the following learning resources did you use for your shifts and the rotation?

Orientation video

Assigned podcast curriculum

Online modules on the learning management system provided (PowerPoint with questions)

AccessEmergencymedicine.com

uptodate.com

A textbook

Other (please specify)

7. How many of each of the following did you do (alone or with assistance)? (0, 1, 2-5, 6-10, >10)

EKG interpretation

Bedside ultrasound

Laceration repair

Lumbar Puncture

Lumbar Puncture 0

Joint reduction or splinting

8. How influential were each of the following in contributing to your learning during your month in the Emergency Department? (1 - Not at all influential, 2 - Slightly influential, 3 - Somewhat influential, 4 - Moderately influential, 5 - Very influential, N/A or did not use/attend)

Emergency medicine attendings

Emergency medicine residents

Nursing staff

Wednesday resident conference session

Online curriculum on the learning management platform

EMS personnel

Other Independent reading

Ultrasound teaching time during teaching shift with resident (put n/a if you did not attend one)

Orientation video

Podcast curriculum assignments

Other (please specify)​​​​​​​

9. How much do you feel your educational goals were met during your rotation?

1 - Not at all

2 - Slightly

3 - Somewhat

4 - Moderately

5 - Very much

Feel free to leave any additional feedback​​​​​​​

10. What are three things that could be done to improve the off-service resident EM rotation?
